# GMOIT: a tool for effective screening of genetically modified crops

**DOI:** 10.1186/s12870-024-05035-2

**Published:** 2024-04-25

**Authors:** Pu Zhou, Xuan Liu, Jingang Liang, Juanli Zhao, Yuqi Zhang, Dongmei Xu, Xiaying Li, Ziyan Chen, Zongyong Shi, Jianhua Gao

**Affiliations:** 1 Hou Ji Laboratory in Shanxi Province, College of Life Sciences, Shanxi Agricultural University, Jinzhong, 030801 Shanxi China; 2 Crops Ecological Environment Security Inspection and Supervision Center (Taiyuan), Ministry of Agriculture and Rural Affairs, Taigu, 030801 Shanxi China; 3 Development Center for Science and Technology, Ministry of Agriculture and Rural Affairs, Beijing, 100025 China

**Keywords:** Genetically modified crops, Transgene detection, Screening strategy, Database, Multi-target plasmids, Biotechnology

## Abstract

**Background:**

Advancement in agricultural biotechnology has resulted in increasing numbers of commercial varieties of genetically modified (GM) crops worldwide. Though several databases on GM crops are available, these databases generally focus on collecting and providing information on transgenic crops rather than on screening strategies. To overcome this, we constructed a novel tool named, Genetically Modified Organisms Identification Tool (GMOIT), designed to integrate basic and genetic information on genetic modification events and detection methods.

**Results:**

At present, data for each element from 118 independent genetic modification events in soybean, maize, canola, and rice were included in the database. Particularly, GMOIT allows users to customize assay ranges and thus obtain the corresponding optimized screening strategies using common elements or specific locations as the detection targets with high flexibility. Using the 118 genetic modification events currently included in GMOIT as the range and algorithm selection results, a “6 + 4” protocol (six exogenous elements and four endogenous reference genes as the detection targets) covering 108 events for the four crops was established. Plasmids pGMOIT-1 and pGMOIT-2 were constructed as positive controls or calibrators in qualitative and quantitative transgene detection.

**Conclusions:**

Our study provides a simple, practical tool for selecting, detecting, and screening strategies for a sustainable and efficient application of genetic modification.

**Supplementary Information:**

The online version contains supplementary material available at 10.1186/s12870-024-05035-2.

## Background

Swift progress of agricultural biotechnology has allowed for increasing numbers of commercial varieties of genetically modified (GM) crops available to producers worldwide. According to the International Service for the Acquisition of Agri-biotech Applications (ISAAA), 190.4 million hectares of GM crops were planted in 2019, an increase of approximately 112-fold relative to that in 1996. With respect to the planted area of specific crops worldwide, 79% of cotton (*Gossypium hirsutum*), 74% of soybean (*Glycine max*), 31% of maize (*Zea mays*), and 27% of canola (*Brassica napus*) are GM varieties [1]. In addition, approximately 25 other GM crops, including alfalfa (*Medicago sativa*), sugar beet (*Beta vulgaris*), and papaya (*Carica papaya*), are grown in different regions of the world, providing diverse choices to consumers. The focus of research on GM crops has also shifted from individual traits, such as insect resistance, herbicide tolerance, and stress resistance, to the simultaneous improvement of complex traits. Furthermore, breeding efforts now aim not just to increase crop yield but to improve both yield and quality [2–5].

Several databases of information on genetically modified organisms (GMO) and GMO assay methods have been established. A case in point, the United Nations Secretariat of the Convention on Biological Diversity has constructed the BCH database (https://bch.cbd.int/), which curates over 1,000 records on GMO crops, including unique identification codes, genetic information, and GM event detection methods [6]. The GMO Approval Database maintained by the ISAAA (http://www.isaaa.org/gmaprovaldatabase/default.asp) records the approval status of GM crops worldwide and contains additional information, such as the GM event name, GM traits, developer, year of planting approval, approval status of processing for food and feed, and a list of national authorizations [7, 8]. Similarly, the GMOMETHODS database (http://gmo-crl.jrc.ec.europa.eu/gmomethods/), developed by the Joint Research Centre of the European Union Reference Laboratory for Genetically Modified Food and Feed (EURL-GMFF), contains quantitative assays for 78 GM crops, qualitative assays for 12 GM crops, several screening elements, and endogenous reference genes. This database includes the name of the GM event, species, references, primer and probe sequences, amplicon lengths, and other information [9]. In turn, JRC GMO-Matrix, a transgene detection and analysis tool developed by the European Union Joint Research Centre (JRC), contains information on a few genetic elements, such as the *35S* promoter and terminator (*P-35S* and *T-35S*) of cauliflower mosaic virus (CaMV) and the terminator of nopaline synthase gene (*T-NOS*) from *Agrobacterium tumefaciens*. Additionally, this tool provides PCR simulation [10, 11]. Finally, the GMDD database (https://gmdd.sjtu.edu.cn/) developed at the GMO Detection Laboratory of Shanghai Jiao Tong University (China) contains information on most approved GM events worldwide and the corresponding detection methods, including basic information, such as the name of the GM event, its unique identification code, trade name, genetic information (e.g., insertion element and published sequences), as well as nucleic acid-based qualitative/quantitative and protein-based detection methods (ELISA and lateral flow immunochromatography assay) [12]. However, all these databases are generally focused on collecting and providing information about transgenic crops rather than on screening strategies.

In this study, a GMO Identification Tool (GMOIT) was created by curating information on 118 independent GM events of four grains and oilseed crops (soybean, maize, canola, and rice), either commercially available or developed in China, and their detection methods. The tool provides flexible and customizable screening strategies for GM elements. Users can set parameters to obtain a wide range of screening strategies for single or multiple crops. The tool can also be used to build element screening schemes covering currently available GM events in these four crops. This is made possible using positive controls or calibrators such as the plasmid constructs developed in the present study, thereby facilitating efficient monitoring and detection of transgenes.

## Results

### Exogenous elements involved in 118 GM events

**Fig. 1:**
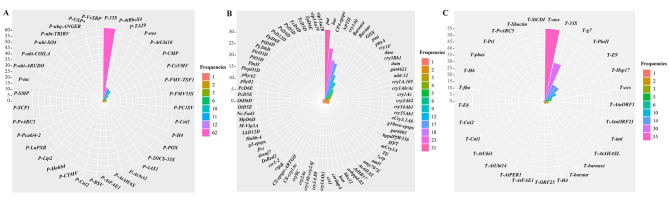
Occurrence frequencies of exogenous elements used in 118 genetic modification (GM) events. (**A**) Promoters; (**B**) Genes; (**C**) Terminators

A total of 115 independent GM events of soybean, rice, canola, and maize were included in the ISAAA database, of which 43 were approved for import in China, while seven were developed in China. In addition, there were three GM events, SHZD32-1, Zhonghuang6106, and Ruifeng125, which were independently developed in China and hold domestic safety certificates (Table S2). There were 36 promoters, 29 terminators, and 78 genes utilized in various frequencies that were involved in these events (Fig. 1 and Table S3). Interestingly, *P-35S*, *pat*, and *T-NOS* represented the highest usage frequencies of GMOIT promoters, genes, and terminators, respectively, across GM crops.

### Generation of screening strategies using GMOIT

**Fig. 2:**
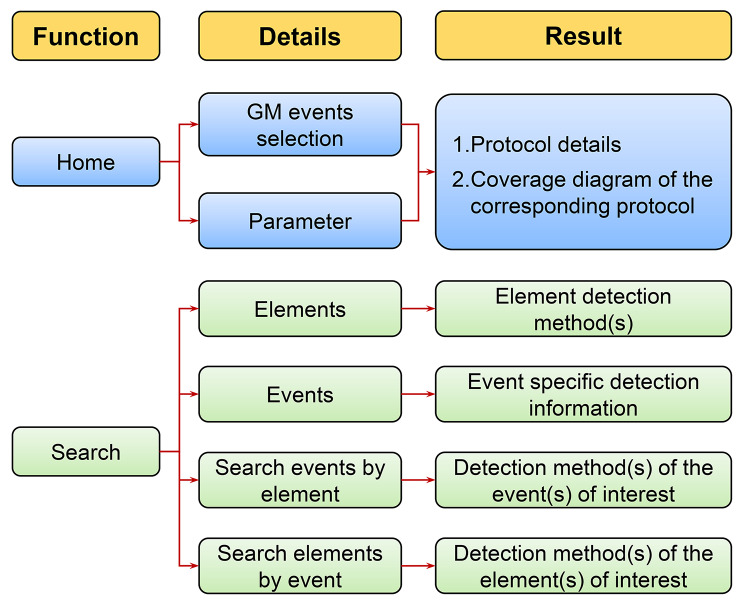
Schematic diagram of the operational GMOIT process

The database performs two major functions: (1) strategy-screening on the Home page and (2) information visualization on the Search page (Fig. 2). The operational procedure is illustrated in Fig. 2.

**Fig. 3:**
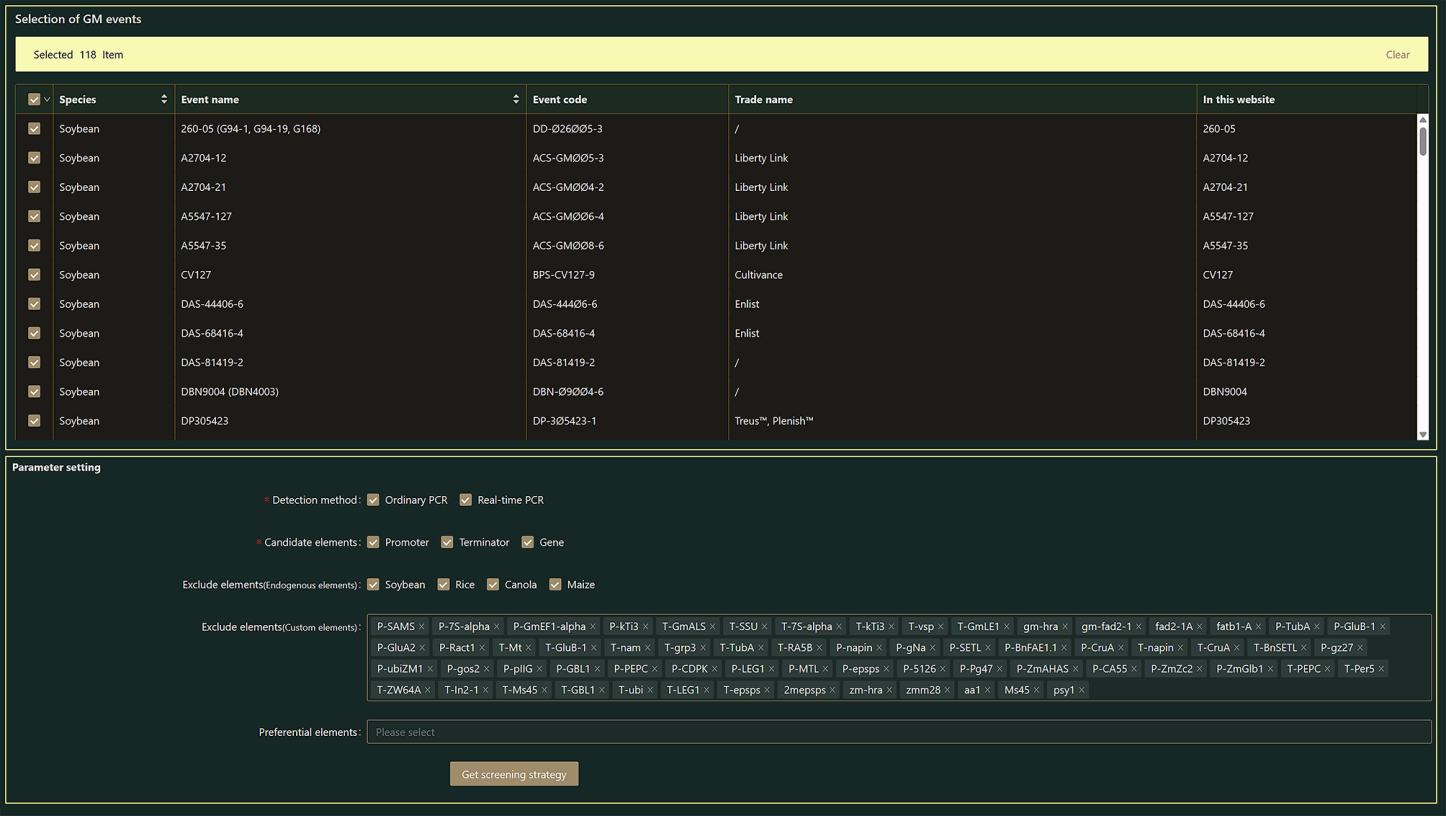
Home page of the GMOIT database

**Screening-strategy search on the Home page.** Four steps have to be completed to obtain the best screening strategy using GMOIT. The GMOIT Home page contains two sections, “Selection of GM events” and “Parameter setting” (Fig. 3).

**Fig. 4:**
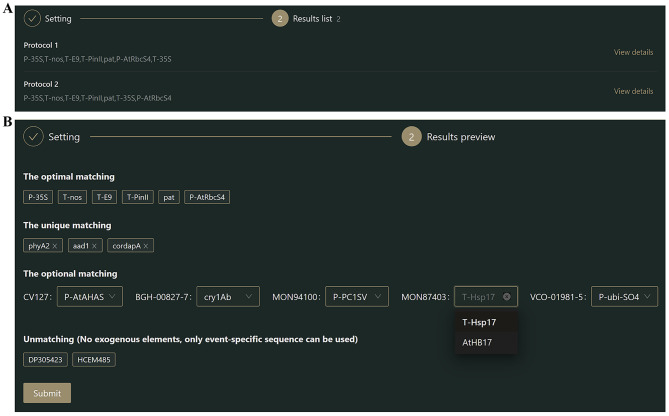
Results list and preview of genetic modification (GM) elements. (**A**) “Results list” provides recommended protocols (screening strategies); (**B**) “Results preview” shows details of the candidate elements for each protocol

**Fig. 5:**
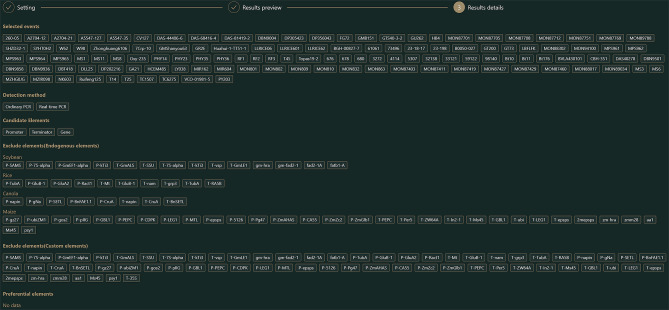
Summary of parameters for the final protocol. Selected events: a list of GM events selected by users is provided. Exclude elements (Endogenous elements): excluded endogenous elements are listed and sorted by crop species. Exclude elements (Custom elements): endogenous elements and excluded elements selected by users are displayed. Preferential elements: preferential elements selected by users are listed

**Fig. 6:**
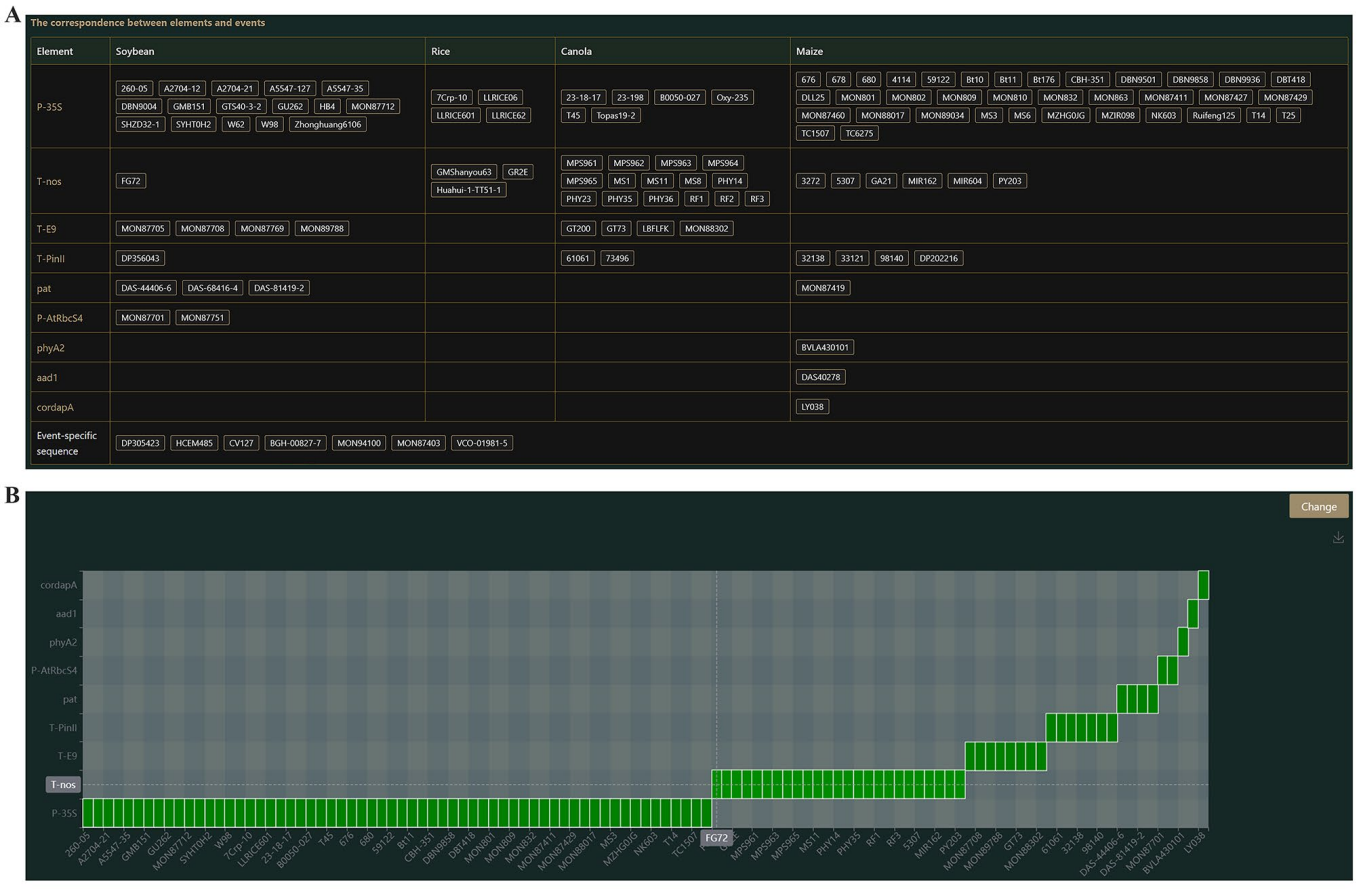
Genetic modification (GM) events and the associated genetic elements events list. (**A**) List of GM events; (**B**) Selected elements associated with GM events

(1) Selection of GM events: The user should select the crop(s) and the corresponding GM event(s) under “Selection of GM events” (Fig. 3). Basic information for each GM event is provided here, including the crop type, name of the GM event, OECD unique identifier, trade name, and the name used in the database. In this study, all 118 GM events curated in the database were considered as targets of interest.

(2) Parameter setting: The user can first select the desired detection method and then the desired candidate element-type(s) (Fig. 3). GMOIT automatically excludes all transgenes or elements originating from the host crop(s) according to user choice. For example, *gm-hra* gene is derived from the non-transgenic soybean genome. When the user selects soybean as the test object, the gene will not be a candidate by default. The user can also customize the element list that includes the member(s) intended to be ruled out (custom elements). Moreover, there are other elements that are potential final targets (preferential elements). This study, for example, added *T-35S* as an additional excluded element. To finish the setting steps, the user needs to click the “Get screening strategy” button to enter the results preview interface (Fig. 4).

(3) Results preview: Multiple candidate protocols are provided in the “Results list” (Fig. 4A). By clicking the “View details” button, the details of the corresponding protocols can be previewed. “The optimal matching” section lists the elements that appear at least twice in the GM events to be tested, and these elements are selected as mandatory targets in the screening strategy by default. “The unique matching” section lists the elements that only appear once. GM events that contain these elements can also be alternatively detected using event-specific sequences, which are generally border sequences between the integrated fragment and insertion site of the genome. Accordingly, the user can decide whether to retain these elements as detection targets. “The optional matching” section lists the elements that either are rarely used as targets for routine detection or occur frequently but with high sequence variation, such as the insecticidal *cry* gene, and are therefore left to user discretion. The “Unmatching” section lists GM events that do not contain exogenous elements and can only be detected by event-specific sequences. Notably, if an element listed in “The unique matching” section or an event listed in “The optional matching” section is removed, the corresponding event will be automatically added to the “Unmatching” section. This study, for example, excluded the uncommon candidates *phyA2*, *aa1*, and *cordapA* from “The unique matching” as well as candidates for the detection of the CV127, BGH-00827-7, MON94100, MON87403, and VCO-01981-5 GM events (Fig. 4B). After making the selections, the user needs to click the “Submit” button to access the “Results details” screen.

(4) Result details: The details of the final protocol are organized here in two sections: (1) General information (Fig. 5) contains user-selected GM events to be tested, detection methods, candidate elements, excluded elements, and preferential elements. (2) The details of the final recommended screening-strategy include a list of correspondence between elements and events (Fig. 6A); this is represented in a schematic diagram of the detection coverage of each element for the GM events (Fig. 6B). In addition, by hovering over a screening element, the user can obtain a tooltip providing detailed information on the detection method for the element, including primer/probe names and sequences, amplicon lengths, data sources, and the suitability of each pair of primer/probe sequences for the corresponding GM insertion for both conventional and real-time fluorescence PCR (Fig. S1).

Using this procedure, a recommended screening strategy including the six exogenous elements *P-35S*, *T-NOS*, *T-pin II*, *T-E9*, *pat*, and *P-AtRbcS4* was obtained (Fig. 6) along with additional information, such as the detection of primer/probe sequences for each screening element (Table S1); this theoretically covers the detection of 108 GM events, i.e., 91.5% of the events included in the database (excluding DP305423, HCEM485, CV127, BGH-00827-7, MON94100, MON87403, VCO-01981-5, DAS40278, BVLA430101, and LY038). By coordinating with the endogenous reference genes viz. *Lectin* (soybean), *zssIIb* (maize), *SPS* (canola), and *Cru A* (rice) of each crop, the “6 + 4” protocol was generated.

**Information visualization on the Search page.** The second major function of the GMOIT database is the ability to retrieve information on detection methods by GM events or exogenous elements (Fig. S2). Four roads lead to Rome. The user would be able to: (1) Directly search the database by entering a given exogenous element (Fig. S2A); (2) Directly search by the GM event of interest (Fig. S2B); (3) Screen the GM events by the given exogenous element, and then obtain information on detection methods of the resultant events (Fig. S2C); (4) Screen the exogenous elements by the given GM event, and then obtain the information on detection methods of the resultant elements (Fig. S2D).

### Affiliated MTPs with the “6 + 4” protocol

**Fig. 7:**
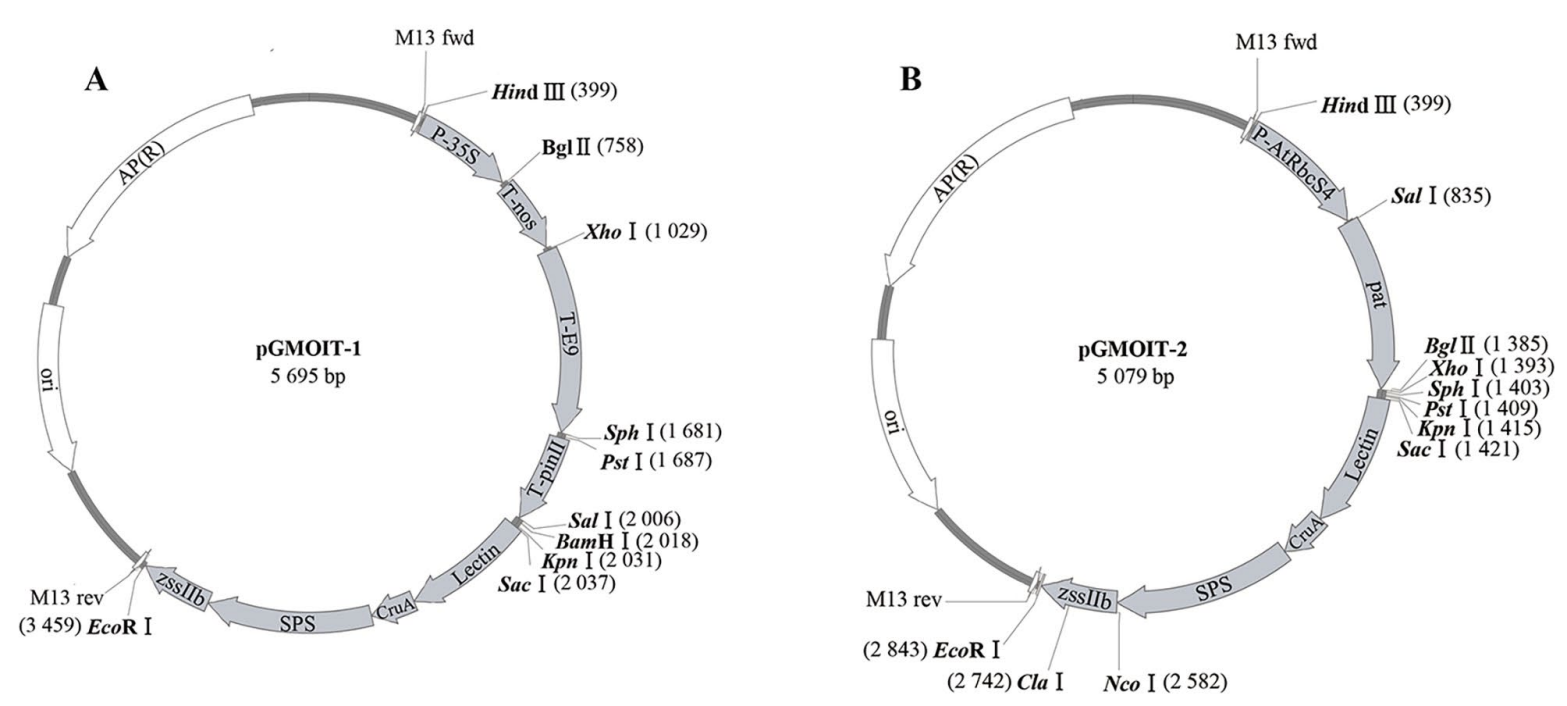
Plasmid constructs used for screening genetic modification (GM) elements. (**A**) pGMOIT-1; (**B**) pGMOIT-2

Based on established screening strategies, a detection coverage of 86.4% (102/118) was achieved for only four elements: *P-35S*, *T-NOS*, *T-E9*, and *T-pin II* (Fig. 6). The other two screening elements, *P-AtRbcS4* and *pat*, covered six GM events. The detection sequences for the first four elements or the latter two elements were concatenated in tandem; they were linked with the detection sequences for the endogenous reference genes viz. *Lectin*, *Cru A*, *SPS*, and *zssIIb* (3066 bp in Fig. S3A and 2450 bp in Fig. S3B) and inserted into the pUC18 plasmid, thereby forming the GM element-screening plasmids pGMOIT-1 (Fig. 7A) and pGMOIT-2 (Fig. 7B). The fragment sizes in the restriction maps of these two plasmids were consistent with expectations (Fig. S4).

### Functional validation of the “6 + 4” protocol and the MTPs

**Validation using conventional PCR.** We first investigated the sensitivity of the selected primers (Table S1) for plasmids pGMOIT-1 and pGMOIT-2. PCR results for different plasmid concentrations showed that the screening elements were successfully amplified with 40 copies or more, thus meeting the requirements for qualitative PCR detection (Fig. S5).

Furthermore, 35 GM events were tested to validate the effectiveness of the protocol for detecting gDNA for GM events. The gDNA of 35 GM events were also used as test samples for validation. The results showed that the corresponding primers were effective in specifically amplifying target sequences in gDNA, and that pGMOIT-1 and pGMOIT-2 can be ideal alternatives for gDNA (Fig. S6).

**Fig. 8:**
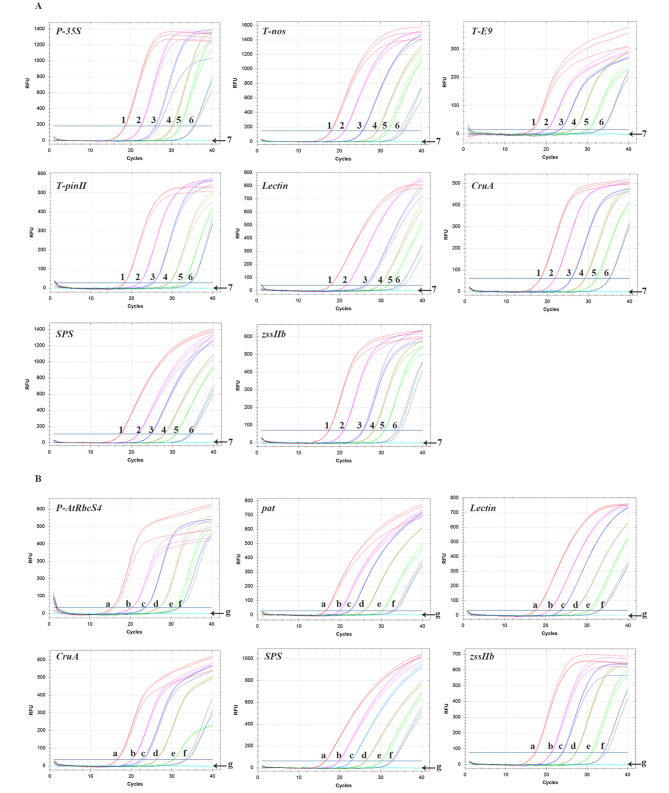
qPCR amplification curve for target elements using plasmids as templates. (**A**) pGMOIT-1 template; 1–6 indicate 5.64 × 10^6^, 5.64 × 10^5^, 5.64 × 10^4^, 5.64 × 10^3^, 5.64 × 10^2^, and 5.64 × 10^1^ copies·µL^− 1^, 7 indicates respectively negative control in which the original pUC18 plasmid was used as template; (**B**) pGMOIT-2 template; a–f indicate 5.51 × 10^6^, 5.51 × 10^5^, 5.51 × 10^4^, 5.51 × 10^3^, 5.51 × 10^2^, and 5.51 × 10^1^ copies·µL^− 1^, respectively, g indicates respectively negative control in which the original pUC18 plasmid was used as template

**Validation using real-time fluorescence PCR.** Real-time fluorescence PCR primers and corresponding TaqMan probes were also tested. qPCR was performed using serially diluted plasmid solutions as templates. The standard curve for each target sequence was plotted based on the relationship between the logarithm of the plasmid template copy-number and the Ct value (Fig. 8 and Fig. S7). The amplification efficiency was 90%–110%, and *R*^2^ values were over 0.98, thus meeting the standard requirements for routine testing [13]. Similarly, qualitative real-time fluorescence PCR was performed using gDNA from samples for the 35 GM events. The amplification results were as expected (Fig. S8).

## Discussion

Most GMO-related databases, such as the BCH database, the GMO Approval Database, and the GMDD, are focused on the compilation of basic information and detection methods for each transgenic event [14, 15]. However, few studies have focused on screening-strategy selection or recommendation. Conversely, the GMOIT database established in the present study fills this gap and provides flexible parameter settings. In recent years, novel techniques such as digital PCR, isothermal nucleic acid amplification, and gene chips have been used for transgene detection [16–18]. Particularly, rapid progress in isothermal nucleic acid amplification technologies, including loop-mediated isothermal amplification [19], recombinase polymerase amplification [20], and rolling circle amplification [21], have provided alternatives to conventional PCR, thereby enabling rapid on-site screening and detection [22, 23]. However, conventional and real-time fluorescence PCR remain the gold standard in transgene detection [24, 25].

Screening every GM event or element is time-consuming and laborious; thus, it is important to set a range of interest and refine the optimal screening protocol [26]. Generally, the operator is most interested in covering the widest possible range with the fewest detection targets [27, 28]. In addition, elements or exogenous genes with the highest usage frequency are preferred. Thus, the primary function of GMOIT is to allow users to rapidly determine the screening strategy that best meets their needs.

The gDNA from positive materials is an important control for transgene detection [29, 30]. However, preparing the positive genetic material derived from GM plants is cumbersome and expensive; fortunately, the use of plasmids containing test target sequences as the positive control can overcome such disadvantages [31]; more importantly, these plasmids show the same usability as gDNA [32–34]. Plasmids can harbor either one segment of the detection target (single-target plasmid) or MTP. For example, Xu et al. [35] constructed a plasmid calibrant, pMON87712, for the GM soybean MON87712. Similarly, Pi et al. [36] constructed the pSOY plasmid to screen five GM events and their derivatives, including A5547-127, DP305423, MON89788, A2704-12, and DP356043. In turn, Park et al. [37] established a screening strategy for 14 GM soybean species and constructed a single target plasmid containing a single element as a positive material. Furthermore, Wen et al. [38] developed a screening strategy for transgenic maize containing nine elements covering 30 GM events in maize, and Li et al. [39] constructed the pBJGMM001 plasmid, whose detection coverage was 96% in all GM rice lines in China. As another example, Ma et al. [40] constructed a positive plasmid pUC18-RICE-screen for the screening and detection of 19 GM rice varieties, and Zhai et al. [41] collected information on 11 GM canola species and established the pYCID-1905 plasmid. Our own group previously constructed the pDDID-1905 plasmid containing sequences specific to 18 GM soybean events approved for import or issuing agricultural GMO safety certificates in China. Particularly, this MTP contains the largest number of soybean GM event-specific sequences [42]. In addition, we have established an “8 + 1” protocol for GM soybean and a companion plasmid, pDDSC-1910, containing nine event-specific sequences viz. *Lectin*, *P-35S*, *T-NOS*, *pat*, *T-E9*, *cry1Ac*, *P-AHAS*, *T-pin II*, and DP305423, covering 29 GM soybean events [27]. However, comprehensive coverage of screening strategies for GM crops and the accompanying MTPs are generally lacking. In this study, we developed a “6 + 4” protocol with positive plasmids for soybean, rice, canola, and maize based on the GMOIT database, which optimizes the detection workload, reduces costs, and supports the routine screening of GM crops.

In summary, GMOIT can produce customizable GM-screening strategies with a user-friendly interface, thus facilitating routine validation and monitoring. Furthermore, the “6 + 4” protocol with the accompanying MTPs, pGMOIT-1, and pGMOIT-2, which cover 108 GM events, can be used directly for routine testing. Moving forward, we will persistently enhance GMOIT by incorporating a broader spectrum of information on crop events.

## Materials and methods

### GMOIT data

Data for soybean, maize, canola, and rice were collected from the ISAAA GMO Approval Database (http://www.isaaa.org/gmaprovaldatabase/default.asp), Biosafety Clearing-House of the Living Modified Organism (LMO) Registry (https://bch.cbd.int/database/lmo-registry/), and the OECD BioTrack Product Database (https://biotrackproductdatabase.oecd.org/byIdentifier.aspx), and organized.

### Strains and plasmids

*Escherichia coli* TOP10 strains and pUC18 plasmids were gifts from the Crop Ecological Environment Security Inspection and Supervision Center (Hefei, China) of the Ministry of Agriculture and Rural Affairs (MARA).

### Samples

Genomic DNA (gDNA) of non-GM soybean, non-GM rice, non-GM canola, non-GM maize, and crop varieties for 32 GM events (DAS-44406-6, DAS-81419-2, DBN9004, DP356043, FG72, GTS40-3-2, MON87701, MON87705, MON87708, MON87751, MON87769, MON89788, SHZD32-1, Zhonghuang6106, Huahui-1-TT51-1, GT73, MS1, Oxy-235, RF1, RF3, T45, Topas 19/2, 59,122, Bt11, Bt176, GA21, MIR604, MON88017, MON89034, NK603, T25, and TC1507) were provided by the Science and Technology Development Center of the MARA. Canola GMO standard 73,496 (ERM-BF434E), maize GMO standards 4114 (ERM-BF439E), and 98,140 (ERM-BF427D) of the European Union Institute for Reference Materials and Measurements (IRMM) were also used.

### Construction of the multi-target plasmid (MTP)

According to the established screening strategy, four target sequences, including *P-35S*, *T-NOS*, *T-E9* (ribulose-1,5-bisphosphate carboxylase small subunit gene terminator), and *T-pin II* (protease inhibitor II gene terminator) were inserted, and the corresponding endonuclease-restriction sites were added at the splice sites of various elements; this was followed by the insertion of targeted sequences of *Lectin*, *zssIIb* (starch synthase IIb), *Cru A* (cruciferin A), and *SPS* (sucrose phosphate synthase) genes. These four genes were used as endogenous reference genes for soybean, maize, canola, and rice, respectively, to determine whether the tested samples contained the corresponding crop species [43, 44]. The spliced sequences were artificially synthesized (Sangon Biotech, Shanghai, China) and cloned into the pUC18 vector between the *Eco*R I and *Hin*d III sites to generate the pGMOIT-1 plasmid. The target sequences of *P-AtRbcS4* (the ribulose-1,5-bisphosphate carboxylase small subunit gene promoter), *pat* (phosphinothricin N-acetyltransferase gene), and the four endogenous reference genes were artificially synthesized (Sangon Biotech) and cloned in the same arrangement described above to generate the pGMOIT-2 plasmid. *Escherichia coli* TOP10 strains containing plasmids pGMOIT-1 or pGMOIT-2 were named T10pGMOIT-1 and T10pGMOIT-2, respectively, and used for plasmid maintenance or expansion. Positive plasmids pGMOIT-1 and pGMOIT-2 were extracted using the Axygen AxyPrep Plasmid Microprep Kit (Thermo Fisher Scientific, Shanghai, China) following manufacturer instructions and validated using enzymatic digestion with different combinations of restriction endonucleases (Thermo Fisher Scientific).

### Validation using conventional PCR

Plasmids pGMOIT-1 and pGMOIT-2 were quantified and serially diluted to form a concentration gradient: 1,000, 100, 40, 20, and 10 copies·µL^-1^, as previously described [27]. Conventional PCR was performed to amplify the targets in the two plasmids using the corresponding primers (Table S1) with the serial dilutions as templates; this was performed to confirm the usability of the two plasmids and to determine the appropriate concentrations of pGMOIT-1 and pGMOIT-2 for conventional PCR assays.

The PCR mixture comprised the following in a total volume of 25 µL: 2.5 µL of 10× PCR Buffer, 1.5 µL of 25 mmol·L^− 1^ MgCl_2_, 2.0 µL of dNTP Mixture (2.5 mmol·L^− 1^ each; TaKaRa Biomedical Technology, Beijing, China), 10 µmol·L^− 1^ forward and reverse primers (1.0 µL each; Sangon Biotech), and 2.0 µL of template DNA; ddH_2_O was added to bring the reaction mixture up to the final volume.

### Validation using real-time fluorescence PCR

Plasmids pGMOIT-1 and pGMOIT-2 were diluted through a gradient (5.64 × 10^6^, 5.64 × 10^5^, 5.64 × 10^4^, 5.64 × 10^3^, 5.64 × 10^2^, and 5.64 × 10^1^ copies·µL^-1^ for pGMOIT-1 and 5.51 × 10^6^, 5.51 × 10^5^, 5.51 × 10^4^, 5.51 × 10^3^, 5.51 × 10^2^, and 5.51 × 10^1^ copies·µL^-1^ for pGMOIT-2) for use as standards in qPCR assays using the corresponding primers and probes (Table S1).

The reaction mixture comprised the following in a total volume of 20 µL: 2.0 µL of 10× PCR Buffer, 2.0 µL of 25 mmol·L^− 1^ MgCl_2_, 1.6 µL of dNTP Mixture (2.5 mmol·L^− 1^ each; TaKaRa Biomedical Technology), 10 µmol·L^− 1^ forward and reverse primers (0.8 µL each; Sangon Biotech), 0.4 µL of 10 µmol·L^− 1^ probe, and 2.0 µL of template DNA; ddH_2_O was added to bring the reaction mixture up to the final volume.

### Electronic supplementary material

Below is the link to the electronic supplementary material.

Supplementary Material 1

Supplementary Material 2

## Data Availability

All the supporting data are included within the article and its additional files. All events contain element information that can be found in the GMO Identification Tool (https://sky.sxau.edu.cn/build/index.html#/).
