# Supplementary material for: GMOIT: a tool for effective screening of genetically modified crops
Source: BMC Plant Biol. 2024 Apr 25;24:329. doi: 10.1186/s12870-024-05035-2 (PMC11044397; doi:10.1186/s12870-024-05035-2)
Supplement: Supplementary file 2 — Supplementary Material 2 [file 12870_2024_5035_MOESM2_ESM.docx]

**
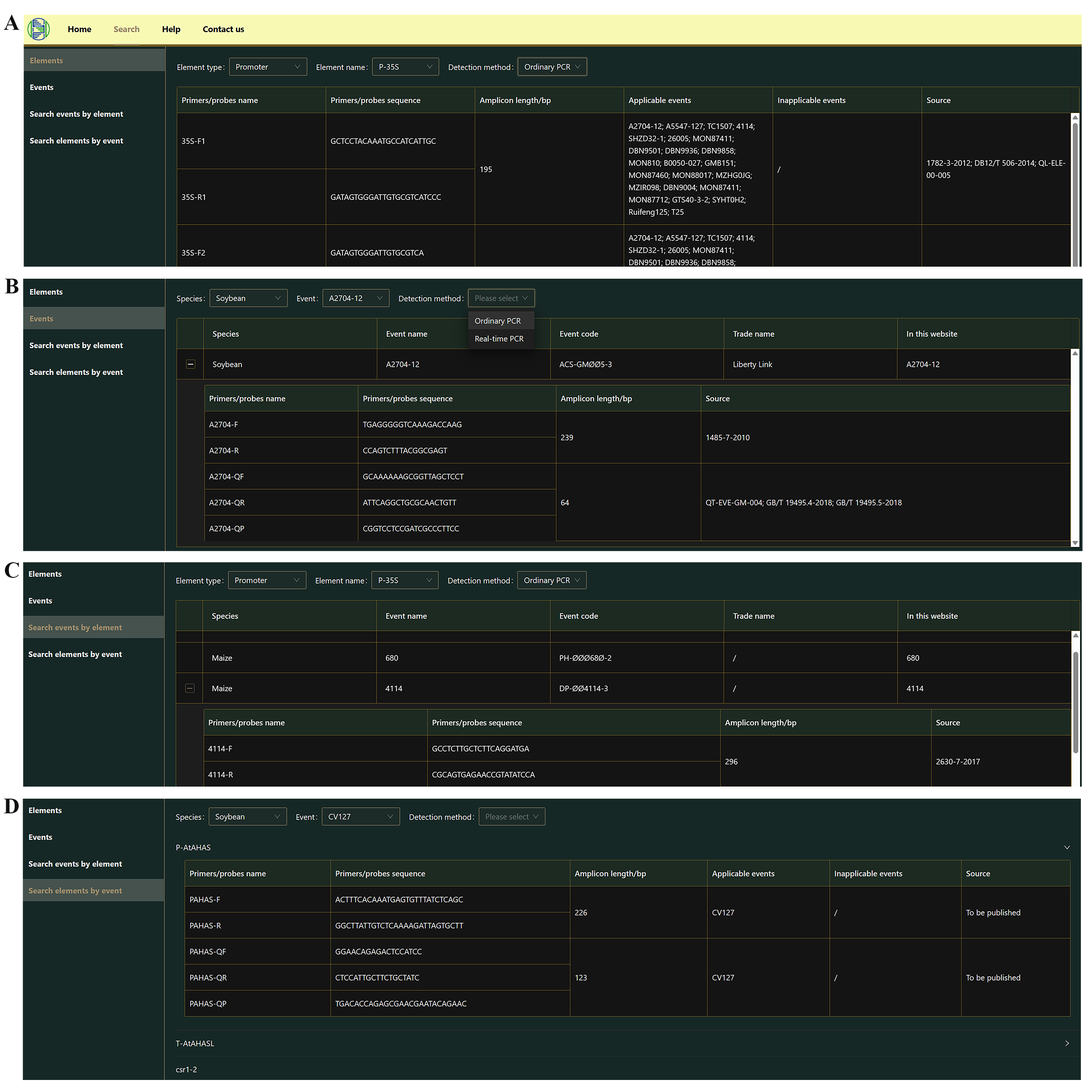
**

**Fig. S2 The interface in Search interfaces menu in GMOIT**

A: Elements module. Users can search the detection method information of the targeted element, including primer/probe name, primer/probe sequences, amplicon length, method source and their applicable events. B: Events module. Users can search the specific-sequence detection method information of the event, including the primer/probe name, primer/probe sequence, amplicon length, and method source. C: Search events by element module. Users can retrieve events that theoretically covered by the selected elements and obtain the specific-sequence detection methods. D: Search elements by event module. Users can search the elements contained in the event selected and obtain the corresponding detection methods information.
