# Supplementary material for: GMOIT: a tool for effective screening of genetically modified crops
Source: BMC Plant Biol. 2024 Apr 25;24:329. doi: 10.1186/s12870-024-05035-2 (PMC11044397; doi:10.1186/s12870-024-05035-2)
Supplement: Supplementary file 1 — Supplementary Material 1 [file 12870_2024_5035_MOESM1_ESM.docx]

**Supplementary Information**

**Fig. S1 The information of the detection methods for each element of interest**

Place the cursor on each element of interest, such as *P-35S*, to display the detection information, including primer/probe name, primer/probe sequences, amplicon length, method source and their applicable events.

**Fig. S2 The interface in Search interfaces menu in GMOIT**

A: Elements module. Users can search the detection method information of the targeted element, including primer/probe name, primer/probe sequences, amplicon length, method source and their applicable events. B: Events module. Users can search the specific-sequence detection method information of the event, including the primer/probe name, primer/probe sequence, amplicon length, and method source, etc. Clicking the plus sign (+) can expand the specific-sequence detection method information of the event of interest (C). D: Search events by element module. Users can retrieve events that theoretically covered by the selected elements and obtain the specific-sequence detection methods by clicking the plus sign (+). E: Search elements by event module. Users can search the elements contained in the event selected and obtain the corresponding detection methods information.

**Fig. S3 Inserted sequence in pGMOIT-1 (A) and pGMOIT-2 (B)**

The names of the targeted elements are listed at the right of the diagram. The detection sequence of each target is included in corresponding box. The main restriction sites are indicated in bold font with underlines.

**Fig. S4 The restriction map of pGMOIT-1 (A) and pGMOIT-2 (B)**

A. The pGMOIT-1 plasmid was digested with restriction enzymes *Hin*d Ⅲ and *Pst* Ⅰ (Lane 1), and then the *Eco*R Ⅰ was added to the reaction system for further digestion (Lane 2). B. The pGMOIT-2 plasmid was digested with restriction enzymes *Hin*d Ⅲ and *Xho* Ⅰ (Lane 1), and then the *Eco*R Ⅰ was added to the reaction system for further digestion (Lane 2). Lane M is the 1 kb plus DNA ladder.

**Fig. S5 Detection sensitivity analysis of pGMOIT-1 (A) and pGMOIT-2 (B) by PCR method**

Lane M: DL2000 DNA Marker (100, 250, 500, 750, 1 000 and 2 000 bp from bottom to top); Lane B: blank control in which ddH_2_O was used as template; Lane "-": negative control in which the original pUC18 plasmid was used as template; Lane "1 000" to "10": the pGMOIT-1 or pGMOIT-2 plasmids with 1 000, 100, 40, 20 or 10 copies of were used as templates, respectively. Each element name was indicated at the bottom right corner and the length of the corresponding amplicon were also showed above the target bands.

**Fig. S6 Functional verification of pGMOIT-1 and pGMOIT-2 for PCR method**

Lane M: DL2000 DNA Marker (100, 250, 500, 750, 1 000 and 2 000 bp from bottom to top); Lane B: blank control in which ddH_2_O was used as template; Lane P^1^ and P^2^: positive control in which the pGMOIT-1 or pGMOIT-2 plasmids were used as template, respectively; Lane S: the corresponding event samples were used as templates; Lane N^1^ ~ N^4^: negative controls in which the non-GM soybean samples, non-GM rice samples, non-GM canola samples or non-GM maize samples were used as templates, respectively. Each element name was indicated at the bottom right corner and the length of the corresponding amplicon were also showed above the target bands.

**Fig. S7 Standard curves and amplification efficiencies of the target elements by qPCR**

**Fig. S8 Functional verification of pGMOIT-1 (A) and pGMOIT-2 (B) for real-time PCR qualitatively**

1: positive control in which the pGMOIT-1 plasmid was used as template. 2: Zhonghuang6106. 3: GTS 40-3-2. 4: MON89034. 5: Oxy-235. 6: NK603. 7: DP356043. 8: GA21. 9: MON88017. 10: FG72. 11: Huahui-1-TT51-1.12: MIR604. 13: MON87701. 14: MON87705. 15: MON89788. 16: MON87708. 17: MON87769. 18: GT73. 19: MON87751. 20: 73496. 21: 98130. 22: 4114. 23: 59122. 24: DBN9004. 25: SHZD32-1. 26: DAS-44406-6. 27: DAS-81419-2. 28: MS1. 29: RF1. 30: RF3. 31: T45. 32: Topas19/2. 33: Bt11. 34: Bt176. 35: T25. 36: TC1507. 37: positive control in which the pGMOIT-2 plasmid was used as template.

**Table S1 Screening targets and the corresponding primers/probes information**

| **Detection**  **target** | **Primer/probe**  **name** | **Primer/probe sequence**  **(5ʹ-3ʹ)** | **Amplicon**  **size**  **/bp** | **Source** | **Selection** | **Applicable event** | **Inapplicable event** |
| --- | --- | --- | --- | --- | --- | --- | --- |
| *P-35S* | 35S-F1 | GCTCCTACAAATGCCATCATTGC | 195 | 1782-3-2012; DB12/T 506-2014; QL-ELE-00-005 | **√** | A2704-12; A5547-127; 4114; 59122; T25; TC1507; B0050-027; DBN9004; DBN9858; MON87429; MZHG0JG; MZIR098; SYHT0H2; MON87419; DAS-44406-6; DAS-68416-4; DAS-81419-2 | / |
|  | 35S-R1 | GATAGTGGGATTGTGCGTCATCCC |  |  |  |  |  |
|  | 35S-F2 | GATAGTGGGATTGTGCGTCA | 195 | SN/T 1195-2003 |  |  |  |
|  | 35S-R2 | GCTCCTACAANTGCCATCA |  |  |  |  |  |
|  | 35S-F3 | CCACGTCTTCAAAGCAAGTGG | 123 | QL-ELE-00-004 |  |  |  |
|  | 35S-R3 | TCCTCTCCAAATGAAATGAACTTCC |  |  |  |  |  |
|  | 35S-F4 | CGACAGTGGTCCCAAAGATG | 159 | Park S-B et al. 2015 (DOI: 10.1016/j.foodcont.2015.01.035) |  |  |  |
|  | 35S-R4 | ATATAGAGGAAGGGTCTTGCG |  |  |  |  |  |
|  | 35S-F5 | GCTCCTACAAATGCCATCA | 195 | QL-ELE-00-001 |  |  |  |
|  | 35S-R5 | GATAGTGGGATTGTGCGTCA |  |  |  |  |  |
|  | 35S-QF1 | CGACAGTGGTCCCAAAGA | 74 | 1782-3-2012 | **√** |  |  |
|  | 35S-QR1 | AAGACGTGGTTGGAACGTCTTC |  |  |  |  |  |
|  | 35S-QP1 | TGGACCCCCACCCACGAGGAGCATC |  |  |  |  |  |
|  | 35S-QF2 | TTCCAACCACGTCTTCAAAGC | 95 | GB/T 38505-2020 |  |  |  |
|  | 35S-QR2 | GGAAGGGTCTTGCGAAGGATA |  |  |  |  |  |
|  | 35S-QP2 | CCACTGACGTAAGGGATGACGCACAATCC |  |  |  |  |  |
|  | 35S-QF3 | GCCTCTGCCGACAGTGGT | 82 | SN/T 1204-2016; GB/T 19495.4-2018; QL-ELE-00-012 |  |  |  |
|  | 35S-QR3 | AAGACGTGGTTGGAACGTCTTC |  |  |  |  |  |
|  | 35S-QP3 | CAAAGATGGACCCCCACCCACG |  |  |  |  |  |
|  | 35S-QF4 | ATTGATGTGATATCTCCACTGACGT | 101 | GB/T 33526-2017 |  |  |  |
|  | 35S-QR4 | CCTCTCCAAATGAAATGAACTTCCT |  |  |  |  |  |
|  | 35S-QP4 | CCCACTATCCTTCGCAAGACCCTTCCT |  |  |  |  |  |
| *T-nos* | NOS-F1 | GAATCCTGTTGCCGGTCTTG | 180 | 1782-3-2012; DB12/T 506-2014; SN/T 1195-2003; QL-ELE-00-006; QL-ELE-00-007 | **√** | DBN9858; DBN9501; DBN9936; B0050-027; MON87460; MON88017; MZHG0JG; MZIR098; FG72; 5307; 3272; MIR604; MIR162; MON87427; GTS40-3-2; SYHT0H2; MS8; RF3; Huahui-TT51-1 | Oxy-235 |
|  | NOS-R1 | TTATCCTAGTTTGCGCGCTA |  |  |  |  |  |
|  | NOS-F2 | CCTGTTGCCGGTCTTGCGAT | 130 | Park S-B et al. 2015 (DOI: 10.1016/j.foodcont.2015.01.035) |  |  |  |
|  | NOS-R2 | TGTATAATTGCGGGACTCTAATCA |  |  |  |  |  |
|  | NOS-F3 | GCATGACGTTATTTATGAGATGGG | 118 | QL-ELE-00-009 |  | DBN9858; DBN9501; DBN9936; B0050-027; MON87460; MON88017; MZHG0JG; MZIR098; FG72; 5307; 3272; MIR604; MIR162; MON87427; GTS40-3-2; SYHT0H2; MS8; RF3; Huahui-TT51-1; Oxy-235 | / |
|  | NOS-R3 | GACACCGCGCGCGATAATTTATCC |  |  |  |  |  |
|  | NOS-QF1 | ATCGTTCAAACATTTGGCA | 165 | 1782-3-2012; GB/T 19495.4-2018; SN/T 1201-2014; GB/T 33526-2017 | **√** | DBN9858; DBN9501; DBN9936; B0050-027; MON87460; MON88017; MZHG0JG; MZIR098; FG72; 5307; 3272; MIR604; MIR162; MON87427; GTS40-3-2; SYHT0H2; MS8; RF3; Huahui-TT51-1 | Oxy-235 |
|  | NOS-QR1 | ATTGCGGGACTCTAATCATA |  |  |  |  |  |
|  | NOS-QP1 | CATCGCAAGACCGGCAACAGG |  |  |  |  |  |
|  | NOS-QF2 | CATGTAATGCATGACGTTATTTATG | 84 | SN/T 1204-2016; QL-ELE-00-011 |  | DBN9858; DBN9501; DBN9936; B0050-027; MON87460; MON88017; MZHG0JG; MZIR098; FG72; 5307; 3272; MIR604; MIR162; MON87427; GTS40-3-2; SYHT0H2; MS8; RF3; Huahui-TT51-1; Oxy-235 | / |
|  | NOS-QR2 | TTGTTTTCTATCGCGTATTAAATGT |  |  |  |  |  |
|  | NOS-QP2 | ATGGGTTTTTATGATTAGAGTCCCGCAA |  |  |  |  |  |
|  | NOS-QF3 | GCATGACGTTATTTATGAGATGGG | 97 | GB/T 38505-2020 |  |  |  |
|  | NOS-QR3 | TCCTAGTTTGCGCGCTATATTT |  |  |  |  |  |
|  | NOS-QP3 | AGAGTCCCGCAATTATACATTTAATACGCG |  |  |  |  |  |
| *T-pinII* | TpinII-F1 | CCTAGACTTGTCCATCTTCTGG | 109 | Park S-B et al. 2015 (DOI: 10.1016/j.foodcont.2015.01.035) |  | 4114; 32138; 33121; 59122; 61061; 73496; 98140; DP202216; DP356043 | / |
|  | TpinII-R1 | CACACAACTTTGATGCCCAC |  |  |  |  |  |
|  | TpinII-F2 | CCATCTTCTGGATTGGCCAACTTAAT | 177 | Park S-B et al. 2016 (DOI: 10.1016/j.foodcont.2016.11.0) |  |  |  |
|  | TpinII-R2 | GACACGTGACATTCATTTAGGAT |  |  |  |  |  |
|  | TpinII-F3 | TGGATTTGGTTAATGAAATGCATCTG | 215 | Zongyong Shi et al. 2021 (DOI: 10.13865/j.cnki.cjbmb.2021.10.1329) |  |  |  |
|  | TpinII-R3 | TGGATTGGCCAACTTAATTAATGTATGAAA |  |  |  |  |  |
|  | TpinII-F4 | GGATGCACACATAGTGACATG | 257 | Designed in this work | **√** |  |  |
|  | TpinII-R4 | GCAAAACACACCTAGACTAGA |  |  |  |  |  |
|  | TpinII-QF1 | GACTTGTCCATCTTCTGGATTGG | 105 | GB/T 38505-2020 | **√** |  |  |
|  | TpinII-QR1 | CACACAACTTTGATGCCCACAT |  |  |  |  |  |
|  | TpinII-QP1 | AGTGATTAGCATGTCACTATGTGTGCATCC |  |  |  |  |  |
|  | TpinII-QF2 | GATAAGAAATATGGATGATCTCTTTCTCTT | 113 | Debode F et al. 2018 (DOI: 10.1007/s12161-018-1203-4) |  |  |  |
|  | TpinII-QR2 | GATGCACACATAGTGACATGCTAATC |  |  |  |  |  |
|  | TpinII-QP2 | TAATTACACATAACACACAACTTTGATGCCCACA |  |  |  |  |  |
|  | TpinII-QF2 | TGACATTCATTTAGGATAAGAAATATGGA | 127 | Self-developed |  |  |  |
|  | TpinII-QR2 | GATGCACACATAGTGACATG |  |  |  |  |  |
|  | TpinII-QP2 | ACACATAACACACAACTTTGATGCCCACATT |  |  |  |  |  |
| *T-E9* | Te9-F1 | GGTCATTAGAGGCCACGATTT | 268 | Li et al. 2015 (DOI: 10.1007/s00216-015-9081-8) |  | DBN9004; LBFLFK; MON87411; MON87705; MON87708; MON87769; MON88302; MON89788 | / |
|  | Te9-R1 | CGAGTATTATGGCATTGGGAAA |  |  |  |  |  |
|  | Te9-F2 | GCATCAGTTTCATTGCGCAC | 168 | Park S-B et al. 2015 (DOI: 10.1016/j.foodcont.2015.01.035) |  | DBN9004; MON87411; MON87705; MON87708; MON87769; MON88302;  MON89788 | LBFLFK |
|  | Te9-R2 | CATTCATTAACTCTTCTCCATCC |  |  |  |  |  |
|  | Te9-F3 | CGCACACACCAGAATCCTACTGA | 285 | Zongyong Shi et al. 2021 (DOI: 10.13865/j.cnki.cjbmb.2021.10.1329) | **√** |  |  |
|  | Te9-R3 | AGGCCACGATTTGACACA |  |  |  |  |  |
|  | Te9-QF1 | TGAGAATGAACAAAAGGACCATATCA | 87 | GB/T 19495.4-2018; QL-ELE-00-024 |  | DBN9004; LBFLFK; MON87411; MON87705; MON87708; MON87769; MON88302; MON89788 | / |
|  | Te9-QR1 | TTTTTATTCGGTTTTCGCTATCG |  |  |  |  |  |
|  | Te9-QP1 | TCATTAACTCTTCTCCATCCATTTCCATTTCACAGT |  |  |  |  |  |
|  | Te9-QF2 | TCTTGTACCATTTGTTGTGCTTGT | 108 | GB/T 38505-2020 | **√** |  |  |
|  | Te9-QR2 | GGACCATATCATTCATTAACTCTTCTCC |  |  |  |  |  |
|  | Te9-QP2 | CGGTTTTCGCTATCGAACTGTGAAATGGAAATGG |  |  |  |  |  |
|  | Te9-QF3 | CGTTCGTCAAGTTCAATGC | 195 | Self-developed |  | DBN9004; MON87411; MON87705; MON87708; MON87769; MON88302;  MON89788 | LBFLFK |
|  | Te9-QR3 | GACCATATCATTCATTAACTCTTCTC |  |  |  |  |  |
|  | Te9-QP3 | CGCACACACCAGAATCCTACTGAGTTTGAG |  |  |  |  |  |
| *pat* | PAT-F1 | GAAGGCTAGGAACGCTTACGA | 262 | 1782-6-2012 |  | A2704-12; A5547-127; 4114; 59122; B0050-027; DBN9004; DBN9858; MON87429; MZHG0JG; MZIR098; SYHT0H2; TC1507; DAS-44406-6; DAS-68416-4; DAS-81419-2; MON87419; T25 | 33121; DP202216 |
|  | PAT-R1 | CCAAAAACCAACATCATGCCA |  |  |  |  |  |
|  | PAT-F2 | GTCGACATGTCTCCGGAGAG | 191 | SN/T 1197-2016 |  | A2704-12; A5547-127; 4114; 59122; DBN9004; DBN9858; MON87429; MZHG0JG; MZIR098; SYHT0H2; TC1507; DAS-44406-6; DAS-68416-4; DAS-81419-2; MON87419; T25 | 33121; DP202216; B0050-027 |
|  | PAT-R2 | GCAACCAACCAAGGGTATC |  |  |  |  |  |
|  | PAT-F3 | CCGGAGAGGAGACCAGTTGAGAT | 227 | Designed in this work | **√** |  |  |
|  | PAT-R3 | TTCCAGGGCCCAGCGTAAG |  |  |  |  |  |
|  | PAT-QF1 | GGAGAGGAGACCAGTTGAGATTAG | 119 | GB/T 38505-2020 |  |  |  |
|  | PAT-QR1 | GTGTTTGTGGCTCTGTCCTAAAG |  |  |  |  |  |
|  | PAT-QP1 | ATCACAAACCGCGGCCATATCAGCTG |  |  |  |  |  |
|  | PAT-QF2 | CGCGGTTTGTGATATCGTTAAC | 108 | QL-ELE-00-025 |  |  |  |
|  | PAT-QR2 | TCTTGCAACCTCTCTAGATCATCAA |  |  |  |  |  |
|  | PAT-QP2 | AGGACAGAGCCACAAACACCACAAGAGTG |  |  |  |  |  |
|  | PAT-QF3 | TTGAGGGTGTTGTGGCTGGTA | 68 | QT-ELE-00-002 |  |  |  |
|  | PAT-QR3 | TGTCCAATCGTAAGCGTTCCT |  |  |  |  |  |
|  | PAT-QP3 | CTTCCAGGGCCCAGCGTAAGCA |  |  |  |  |  |
|  | PAT-QF4 | GTCGACATGTCTCCGGAGAG | 191 | GB/T 19495.4-2018; SN/T 1197-2016; SN/T 2705-2010; SN/T 1201-2014; SN/T 1204-2016; To be published | **√** |  |  |
|  | PAT-QR4 | GCAACCAACCAAGGGTATC |  |  |  |  |  |
|  | PAT-QP4 | TGGCCGCGGTTTGTGATATCGTTAA |  |  |  |  |  |
| *P-AtRbcS4* | RbcS4-F1 | AATGAGCAAGCACCACTCCA | 133 | Park S-B et al. 2015 (DOI: 10.1016/j.foodcont.2015.01.035) | **√** | MON87701; MON87751; MS8; RF3 | / |
|  | RbcS4-R1 | AAGGCCTAAGGAGAGGTGTT |  |  |  |  |  |
|  | RbcS4-QF1 | CCACTCCACCATCACACAATTTC | 112 | GB/T 38505-2020 | **√** |  |  |
|  | RbcS4-QR1 | GGAGAGGTGTTGAGACCCTTATC |  |  |  |  |  |
|  | RbcS4-QP1 | ACGTGGCATTATTCCAGCGGTTCAAGCC |  |  |  |  |  |
|  | RbcS4-QF2 | GGCCTAAGGAGAGGTGTGGAGA | 95 | GB/T 19495.4-2018 |  |  |  |
|  | RbcS4-QR2 | CTCATAGATAACGATAAGATTCATGGAATT |  |  |  |  |  |
|  | RbcS4-QP2 | CCTTATCGGCTTGAACCGCTGGAATAA |  |  |  |  |  |
| *Lectin* | Lectin-F1 | GCCCTCTACTCCACCCCCATCC | 118 | QL-TAX-GM-002 |  | / | / |
|  | Lectin-R1 | GCCCATCTGCAAGCCTTTTTGTG |  |  |  |  |  |
|  | Lectin-F2 | GGGTGAGGATAGGGTTCTCTG | 210 | 2031-8-2013 | **√** |  |  |
|  | Lectin-R2 | GCGATCGAGTAGTGAGAGTCG |  |  |  |  |  |
|  | Lectin-QF1 | GCCCTCTACTCCACCCCCA | 118 | 2031-8-2013; QT-TAX-GM-007 | **√** |  |  |
|  | Lectin-QR1 | GCCCATCTGCAAGCCTTTTT |  |  |  |  |  |
|  | Lectin-QP1 | AGCTTCGCCGCTTCCTTCAACTTCAC |  |  |  |  |  |
|  | Lectin-QF2 | CTTTCTCGCACCAATTGACA | 102 | QT-TAX-GM-003 |  |  |  |
|  | Lectin-QR2 | TCAAACTCAACAGCGACGAC |  |  |  |  |  |
|  | Lectin-QP2 | CCACAAACACATGCAGGTTATCTTGG |  |  |  |  |  |
|  | Lectin-QF3 | CCAGCTTCGCCGCTTCCTTC | 74 | GB/T19495.5-2018; QT-TAX-GM-002 |  |  |  |
|  | Lectin-QR3 | GAAGGCAAGCCCATCTGCAAGCC |  |  |  |  |  |
|  | Lectin-QP3 | CTTCACCTTCTATGCCCCTGACAC |  |  |  |  |  |
|  | Lectin-QF4 | TCCACCCCCATCCACATTT | 81 | QT-TAX-GM-001 |  |  |  |
|  | Lectin-QR4 | GGCATAGAAGGTGAAGTTGAAGGA |  |  |  |  |  |
|  | Lectin-QP4 | AACCGGTAGCGTTGCCAGCTTCG |  |  |  |  |  |
|  | Lectin-QF5 | TGGTCGCGCCCTCTACTC | 70 | QT-TAX-GM-004 |  |  |  |
|  | Lectin-QR5 | GGCGAAGCTGGCAACG |  |  |  |  |  |
|  | Lectin-QP5 | CTACCGGTTTCTTTGTCCCAAATGTGGAT |  |  |  |  |  |
| *Cru A* | CruA-F1 | GGCCAGGGCTTCCGTGAT | 151 | 2031-9-2013 | **√** | / | / |
|  | CruA-R1 | CTGGTGGCTGGCTAAATCGA |  |  |  |  |  |
|  | CruA-QF1 | GGCCAGGGTTTCCGTGAT | 101 | GB/T 19495.4-2018; 2031-9-2013 | **√** |  |  |
|  | CruA-QR1 | CCGTCGTTGTAGAACCATTGG |  |  |  |  |  |
|  | CruA-QP1 | AGTCCTTATGTGCTCCACTTTCTGGTGCA |  |  |  |  |  |
| *SPS* | SPS-F1 | TTGCGCCTGAACGGATAT | 277 | QL-TAX-OS-006 |  | / | / |
|  | SPS-R1 | GGAGAAGCACTGGACGAGG |  |  |  |  |  |
|  | SPS-F2 | ATCTGTTTACTCGTCAAGTGTCATCTC | 287 | 1861-1-2012 | **√** |  |  |
|  | SPS-R2 | GCCATGGATTACATATGGCAAGA |  |  |  |  |  |
|  | SPS-QF1 | TTGCGCCTGAACGGATAT | 81 | 1861-1-2012; QT-TAX-OS-003; GB/T19495.5-2018 | **√** |  |  |
|  | SPS-QR1 | CGGTTGATCTTTTCGGGATG |  |  |  |  |  |
|  | SPS-QP1 | TCCGAGCCGTCCGTGCGTC |  |  |  |  |  |
| *zssⅡb* | zssⅡb-F1 | CTCCCAATCCTTTGACATCTGC | 151 | 1861-3-2012 | **√** | / | / |
|  | zssⅡb-R1 | TCGATTTCTCTCTTGGTGACAGG |  |  |  |  |  |
|  | zssⅡb-QF1 | CGGTGGATGCTAAGGCTGATG | 88 | 1861-3-2012 | **√** |  |  |
|  | zssⅡb-QR1 | AAAGGGCCAGGTTCATTATCCTC |  |  |  |  |  |
|  | zssⅡb-QP1 | AGATGCGGCGGCGAGTGCTCCTTA |  |  |  |  |  |
|  | zssⅡb-QF2 | CTCCCAATCCTTTGACATCTGC | 151 | QT-TAX-ZM-006; GB/T19495.4-2018 |  |  |  |
|  | zssⅡb-QR2 | TCGATTTCTCTCTTGGTGACAGG |  |  |  |  |  |
|  | zssⅡb-QP2 | AGCAAAGTCAGAGCGCTGCAATGCA |  |  |  |  |  |

**Table S2 List of GM events in GMOIT**

| **Crops** | **Approvals** | **Numbers** | **GM Events** |
| --- | --- | --- | --- |
| Soybean | Approved for import | 17 | A2704-12, A5547-127, CV127, DAS-44406-6, DAS-81419-2, DP305423, DP356043, FG72, GTS 40-3-2, MON87701, MON87705, MON87708, MON87769, MON89788, MON87751, SYHT0H2, DBN9004 |
|  | Unapproved for import | 10 | A2704-21, A5547-35, DAS-68416-4, 260-05, GU262, HB4, MON87712, W62, W98, GMB151 |
|  | Issued the agricultural safety certificate  for GMOs  (Domestically developed) | 2 | SHZD32-1, Zhonghuang6106 |
| Rice | Approved for import | 0 | / |
|  | Unapproved for import | 8 | 7Crp#10, BGH-00827-7, GR2E, LLRICE06, LLRICE601, LLRICE62, GM Shanyou 63, Huahui-1-TT51-1 |
| Canola | Approved for import | 6 | GT73, MON88302, Oxy-235, RF3, T45, Topas19/2 |
|  | Unapproved for import | 22 | 61061, 73496, 23-18-17, 23-198, B0050-027, GT200, LBFLFK, MPS961, MPS962, MPS963, MPS964, MPS965, MS1, MS11, MS8, PHY14, PHY23, PHY35, PHY36, RF1, RF2, MON94100 |
| Maize | Approved for import | 21 | 3272, 4114, 5307, 59122, Bt11, Bt176, DAS40278, GA21, MIR162, MIR604, MON810, MON863, MON87411, MON87427, MON87460, MON88017, MON89034, MZIR098, NK603, T25, TC1507 |
|  | Unapproved for import | 31 | 676, 678, 680, 32138, 33121, 98140, Bt10, CBH-351, DBT418, DLL25, DP202216, HCEM485, LY038, MON801, MON802, MON809, MON832, MON87403, MON87419, MON87429, MS3, MS6, MZHG0JG, T14, TC6275, VCO-01981-5, PY203, BVLA430101, DBN9858, DBN9936, DBN9501 |
|  | Issued the agricultural safety certificate for GMOs  (Domestically developed) | 1 | Ruifeng125 |

**Table S3 Occurrence frequencies of exogenous elements**

| **Type** | **Frequencies** | **Element name** |
| --- | --- | --- |
| Promoter | 62 | *P-35S* |
|  | 12 | *P-AtRbcS4* |
|  | 11 | *P-TA29* |
|  | 10 | *P-nos* |
|  | 6 | *P-AtUbi10* |
|  | 3 | *P-FMV-TSF1、P-FMV35S、P-CMP、P-PC1SV、P-CsVMV* |
|  | 2 | *P-H4、P-POX、P-Cnl1* |
|  | 1 | *P-SCP1、P-4AS1、P-2OCS-35S、P-ubi-TRIRV、P-ubi-COILA、P-ubi-ARUDO、P-ubi-SO4、P-SMP、P-AtAHAS、P-Hahb4、P-AtAct2、P-CYMV、P-tac、P-ubq-ANGER、P-BSV、P-Cnl2、P-AtFAE1、P-Ltp2、P-USP+、P-VcSBP、P-LuPXR、P-PvARC5、P-Pcubi4-2* |
| Gene | 31 | *Pat* |
|  | 23 | *Bar* |
|  | 18 | *CP4-epsps* |
|  | 17 | *NPTⅡ* |
|  | 12 | *cry1Ab* |
|  | 9 | *Barstar、Barnase* |
|  | 6 | *GOX、pmi* |
|  | 5 | *phyA* |
|  | 4 | *cry1F、dmo* |
|  | 3 | *gat4621、cry3Bb1、dam* |
|  | 2 | *cry1Ac、gat4601、g10evo-epsps、cry1A.105、cry1Ab/Ac、eCry3.1Ab、cry2Ab2、cry34Ab1、cry35Ab1、add-12、hppdPfW336、HPT、TE、mCry3A* |
|  | 1 | *g2-epsps、CS-epsps-ARTGO、CS-cry1Ac、cry1Ab-cry2Aj、cry9C、cry2Ae、cry1A.88、cry14Ab1、Vip3Aa19、Vip3Aa20、M-Vip3A、csr1-2、bbx32、Nc.Fad3、Pj.D6D、avhppd-03、Hahb-4、phyA2、amy797E、DsRed2、dvsnf7、cordapA、ft-t、AtHB17、PinII、aad1、bxn、7crp、crt1、cspB、LkD12D、PicpO3D、MpD6D、PcD6E、PsD5D、PcD5E、PsD4D、PpD6E、TcD5D、OtD6D、TpD6E、PsD12D、PirO3D、PiO3D、TcD4D、PlD4D、OtD5E、AtAHAS、phy02* |
| Terminators | 55 | *T-nos* |
|  | 30 | *T-35S* |
|  | 14 | *T-PinII、T-g7* |
|  | 10 | *T-E9* |
|  | 6 | *T-Hsp17* |
|  | 5 | *T-ocs* |
|  | 3 | *T-AtuORF23、T-AtuORF1、T-tml* |
|  | 2 | *T-AtAHASL、T-H4、T-barstar、T-barnase、T-ORF25* |
|  | 1 | *T-E6、T-Pt1、T-H6、T-phas、T-Cnl1、T-Cnl2、T-AtUbi3、T-AtUbi14、T-StCDI、T-AtPER1、T-PvARC5、T-AtFAE1、T-Sbactin、T-fba* |
